# Simultaneous Correlative Interferometer Technique for Direction Finding of Signal Sources

**DOI:** 10.3390/s23218938

**Published:** 2023-11-02

**Authors:** Minkyu Oh, Young-Seok Lee, In-Ki Lee, Bang Chul Jung

**Affiliations:** 1 Department of Electronics Engineering, Chungnam National University, Daejeon 34134, Republic of Korea; minkyuoh@o.cnu.ac.kr (M.O.); yslee@o.cnu.ac.kr (Y.-S.L.); 2 Satellite Communication Infra Research Section, Electronics and Telecommunications Research Institute, Daejeon 34129, Republic of Korea; popularity1@etri.re.kr

**Keywords:** 6G, wireless positioning, Direction of Arrival (DOA), direction finding, array antenna, Uniform Circular Array (UCA), Correlative Interferometer (CI), Root Mean Squared Error (RMSE)

## Abstract

In this paper, we propose a novel simultaneous Correlative Interferometer (CI) technique that elaborately estimates the Direction of Arrival (DOA) of multiple source signals incident on an antenna array. The basic idea of the proposed technique is that the antenna-array-based receiver compares the phase of the received signal with one of the candidates at each time sample and jointly exploits these multiple time samples to estimate the DOAs of multiple signal sources. The proposed simultaneous CI-based DOA estimation technique collectively utilizes multiple time-domain samples and can be regarded as a generalized version of the conventional CI algorithm for the case of multiple time-domain samples. We first thoroughly review the conventional CI algorithm to comprehensively explain the procedure of the direction-finding algorithm that adopts the phase information of received signals. We also discuss several technical issues of conventional CI-based DOA estimation techniques that are originally proposed for the case of a single time-domain sample. Then, we propose a simultaneous CI-based DOA estimation technique with multi-sample diversity as a novel solution for the case of multiple time-domain samples. We clearly compare the proposed simultaneous CI technique with the conventional CI technique and we compare the existing Multiple Signal Classification (MUSIC)-based DOA estimation technique with the conventional CI-based technique by using the DOA spectrum as well. To the best of our knowledge, the simultaneous CI-based DOA estimation technique that effectively utilizes the characteristics of multiple signal sources over multiple time-domain samples has not been reported in the literature. Through extensive computer simulations, we show that the proposed simultaneous CI technique significantly outperforms both the conventional CI technique in terms of DOA estimation even in harsh environments and with various antenna array structures. It is worth noting that the proposed simultaneous CI technique results in much better performance than the classical MUSIC algorithm, which is one of the most representative subspace-based DOA estimation techniques.

## 1. Introduction

Wireless positioning and sensing are expected to become emerging technologies as new service applications and scenarios for the next-generation mobile communication systems, such as autonomous driving, Industrial Internet of Things (IIoT), remote robots, etc. [1]. Therein, direction finding of (multiple) signal sources has been substantially developed and has become an important part of recent and ongoing standards, including the 3rd Generation Partnership Project (3GPP) [2] and Institute of Electrical and Electronics Engineers (IEEE) [3].

Recently, Direction of Arrival (DOA) estimation techniques using array antennas have attracted much attention for achieving high resolution and robust performance in direction finding for various purposes [4,5,6]. In [4], DOA estimation was used for the Global Navigation Satellite System (GNSS) receiver to detect a spoofing signal, which is a counterfeit GNSS-like signal for deception. In [5], a DOA estimation method with improved accuracy was proposed with a reconfigurable intelligent surface in an environment considering the positional perturbation of an Unmanned Aerial Vehicle (UAV) swarm. In [6], considering Vehicle-to-Everything (V2X) communication, a DOA-based localization method with large-scale arrays was investigated to guarantee safety and security.

In addition, many array-antenna-based DOA estimation algorithms for detecting directions of multiple signal sources have been investigated over the past few decades. Actually, when an array-antenna-based receiver estimates the DOA of multiple signal sources, the Maximum Likelihood Estimator (MLE) is known to be the optimal DOA estimation algorithm [7]. However, the MLE-based DOA estimation requires an extremely high computational complexity, and it may not be feasible to assume that the covariance of the noise component and the non-desired signal components must be known. On the other hand, Multiple Signal Classification (MUSIC) is one of the well-known practical DOA estimation techniques with an array antenna by exploiting the orthogonality between the subspaces of signal components and noise components in the covariance matrix of the received signal [8]. In [9], a two-dimensional DOA estimation technique with an imperfect aped antenna array was proposed, where two uniform linear arrays are not perpendicular to each other. In particular, two different DOA estimation techniques were considered, which are based on MUSIC and Iterative Maximum Likelihood Calibration (IMLC), respectively. However, in the practical MUSIC algorithm, calculating the covariance of the received signal should be performed by collecting a large number of (time-domain) received signal samples, and it also requires high-complexity computations such as eigenvalue decomposition. In addition, parameter-based DOA estimation algorithms such as root-MUSIC [10] and Estimation of Signal Parameters via Rotational Invariance Techniques (ESPRIT) [11] have been frequently used to reduce the time and computational load of the existing MUSIC where the receiver estimates the DOAs by exhaustively exploring each candidate direction. However, since these algorithms use the phase difference between equally spaced array antennas, they can be applied to a certain array architecture. On the other hand, Compressed Sensing (CS)-based DOA algorithms using sparsity between incident directions are also being actively studied [12,13,14]. In [12], a robust phase-ambiguity-immune DOA estimation algorithm was proposed, which does not require the phase correction at the multi-channel receiver and enables the DOA estimation for multiple sources in a single-channel system. In [13], a novel CS-based DOA estimation technique was proposed for efficiently detecting GNSS spoofing attacks with a small number of time samples. In [14], another CS-based DOA estimation algorithm was proposed, which outperforms the conventional subspace-based DOA estimation method even in a lower number of array elements and in severely noisy environments. Moreover, there are several related studies adopting machine-learning-based DOA estimation [15,16,17,18], but these techniques may induce significant implementation and operation complexity due to (neural network) training in advance, and thus they may not be feasible to be applied for real-time dynamic DOA estimation systems.

DOA estimation extracting spatial characteristics through the phase of the received signal directly, called Correlative Interferometer (CI), is also being actively investigated in the literature thanks to its good performance, computational efficiency, high flexibility, and low-complexity implementation [19,20,21,22,23,24,25]. In [19], an antenna-array cascade system was proposed to improve DOA estimation performance by resolving DOA ambiguity due to the small number of antenna elements. In [20], a low-complexity CI algorithm using a modified cost function for real-time two-dimensional DOA estimation with uniform circular array (UCA) was proposed. In [21], a time-modulated array-based DOA estimation technique was proposed for improving the accuracy of direction finding. Specifically, the CI algorithm is a greedy DOA estimation technique that selects the most likely phase candidate using the difference between the phase of the received signal and all candidate phases, extracting the directional information of the selected phase. Herein, various cost functions can be used as the criteria for selecting the most plausible phase candidate. In [22], three cost functions for the CI algorithm were presented and verified by comparing the performance difference between each cost function and the Cramér–Rao bound. The CI algorithm was already used for direction finding with ULA [23] and UCA [24]. A modified CI using element-radiated power pattern to estimate the DOA of multiple signals with UCA was presented in [25]. However, most existing studies mainly require a single source environment or a high Signal-to-Noise Ratio (SNR) assumption. Furthermore, the CI algorithm has rarely been presented on how it works when receiving multiple time-domain received signal samples. If the receiver collects a few samples and jointly exploits the spatial features of the received signal within each sample, it might achieve improved DOA estimation performance while having higher resolution. To the best of our knowledge, the extended CI algorithms that can properly utilize the spatial characteristics of signal sources in multiple samples have not been reported in the literature.

In this paper, we propose a novel simultaneous CI technique that extends the conventional CI algorithm for the direction finding of multiple signal sources by using multiple time-domain received signal samples. First, we investigate the conventional CI algorithm to address the direction-finding mechanism using phase difference. Then, we identify that the DOA estimation can be inaccurate due to the effect of the noise component on the signal phase when applying the CI algorithm to a single sample, especially in the low-SNR region. Finally, we present a novel simultaneous CI that can jointly explore the spatial characteristics of the received signal in each time-domain sample to guarantee robust DOA estimation performance even at low-SNR regions. In addition, it is worth noting that we propose the simultaneous CI technique from a signal processing perspective. In general, the experiments with hardware implementation of direction-finding techniques require significant time and cost. Hence, most studies utilize computer simulations to validate their proposed techniques [26,27,28,29]. Hence, we also validate our proposed simultaneous CI technique through extensive computer simulations in this paper. We show that the simultaneous CI technique elaborately estimates the DOA of multiple signal sources at the low-SNR region and significantly outperforms the conventional MUSIC and CI algorithm for all SNR regions through extensive computer simulations.

The rest of the paper is organized as follows:

- In Section 2, the system model and assumptions we consider in this paper are described.
- In Section 3 and Section 4, we review the conventional CI-based DOA estimation technique and propose our simultaneous CI-based DOA estimation technique in detail, respectively.
- In Section 6, we compare the conventional MUSIC and CI methods with a normalized DOA spectrum to clearly explain where the performance difference between them comes from.

The main contributions of this paper are summarized as follows:

- We propose a novel simultaneous CI technique exploiting multiple time samples to improve DOA estimation performance, which can be regarded as a generalized algorithm of the conventional CI method.
- We show that the proposed technique with various array structures significantly outperforms the conventional DOA estimation methods, especially in harsh estimation environments, through extensive computer simulations.

## 2. System Model

**Figure 1:**
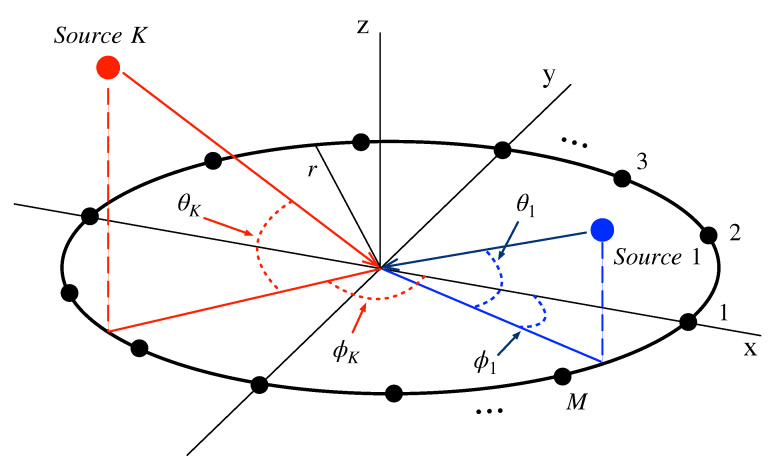
Structure of the simultaneous CI system for 2D direction estimation.

Without loss of generality, we consider the origin-centered UCA-based receiver consisting of *M* antenna elements, which tries to estimate DOAs of *K* signal sources as illustrated in Figure 1.

The system model of this paper deals with generalized steering vector and received signal model, and thus it can be easily extended to any other array structures. In addition, it is assumed that all signal sources exist in Line of Sight (LoS) to the receiver; multipath signals are negligibly small or absent as in many related studies. Then, the received signal, $y(\in C^{M})$, at the UCA receiver is given by

(1) y=Ax+w,

where $A(\in C^{M\times K})$ denotes the matrix concatenating the steering vectors of each incident signal source, i.e., $A=\left[a\left(\gamma_{1}\right)\cdots a\left(\gamma_{k}\right)\cdots a\left(\gamma_{K}\right)\right]$. Herein, a column of $a\left(\gamma_{k}\right)\left(\in C^{M}\right)$ indicates the steering vector of k∈{1,⋯,K}-th source. When considering a UCA as a receiving array antenna, the directional information, $\gamma_{k}=\left\{\phi_{k},\theta_{k}\right\}$, represents a set consisting of the azimuth angle $\phi_{k}\left(\in \left[-\pi ,\pi \right]\right)$ and the elevation angle $\theta_{k}\left(\in \left[0,\pi /2\right]\right)$ of the *k*-th signal source. In addition, we assume that the distance between all signal sources and the receiver is far enough and thus the far-field assumption is valid. Therefore, only the position vector of each array element and signal directional vector are used to effectively reflect the distance difference that occurs when the plane wave reaches each antenna to the phase under the far-field assumption. Then, the m∈{1,⋯,M}-th element of the steering vector $a_{m}\left(\gamma_{k}\right)$ is defined as

(2) $$a_{m}\left(\gamma_{k}\right)=e^{-j\frac{2\pi }{\lambda }p_{m}\;\cdot \;s\left(\gamma_{k}\right)}$$

where λ means the wavelength, $p_{m}\left(\in R^{3}\right)$ is the position vector of *m*-th element in Cartesian coordinate, and $s\left(\gamma_{k}\right)\left(\in R^{3}\right)$ is the directional vector of *k*-th signal source given by

(3) $$s\left(\gamma_{k}\right)=-{\left[\cos\phi_{k}\cos\theta_{k},\;\sin\phi_{k}\cos\theta_{k},\;\sin\theta_{k}\right]}^{T}.$$

Also, $x(\in R^{K})$ in (1) means the received signal vector from *K* sources. In this paper, to evaluate the effect of the direction-finding performance caused only by additive noise, it is assumed that both Doppler frequency and carrier frequency are compensated in advance. In other words, $x_{k}$, the *k*-th element of x, represents the signal power of *k*-th signal source, i.e., $x_{k}=\sqrt{P_{k}}$, where $P_{k}$ denotes the power of *k*-th signal source. Finally, $w(\in C^{M})$ denotes the Additive White Gaussian Noise (AWGN) vector, and it is assumed that $w∼\mathrm{CN}(0,\sigma^{2}I_{M})$.

## 3. Correlative Interferometer Method

In this section, we first investigate the conventional CI algorithm to address the direction-finding mechanism through the phase difference. Then, we specify some problems with the existing CI algorithm that can arise when using only a single sample.

The CI algorithm can simply be performed by measuring the phase of the received signal incident on each antenna element and comparing it with the candidate phases. Specifically, if the signal received by the *m*-th antenna element is $y_{m}$, the phase of $y_{m}$ is denoted as $r_{m}$ in this paper, i.e., $r_{m}=∠y_{m}$. Further, assuming that the array type and position vector are known, from (2) and (3), the candidate phase $c_{m}\left(\hat{\gamma }\right)$ for an arbitrary directional information $\hat{\gamma }=\left\{\hat{\phi },\hat{\theta }\right\}$ can be defined as

(4) $$c_{m}\left(\hat{\gamma }\right)=-\frac{2\pi }{\lambda }p_{m}\cdot s\left(\hat{\gamma }\right),$$

where the candidate directional information $\hat{\gamma }$, in this paper, is defined as an element set in a dictionary tensor A. That is, A has candidate sets representing pairs of the candidate azimuth and elevation angles as its elements, and, for a total range of *A*, it can be expressed as

(5) $$A≜\{\left\{{\hat{\phi }}_{1},{\hat{\theta }}_{1}\right\},\;\left\{{\hat{\phi }}_{2},{\hat{\theta }}_{2}\right\},\cdots ,\left\{{\hat{\phi }}_{A},{\hat{\theta }}_{A}\right\}\}.$$

For example, considering 1-degree interval resolution for both candidate azimuth angle $\hat{\phi }\in \left(0^{\circ },360^{\circ }\right]$ and elevation angle $\hat{\theta }\in \left[0^{\circ },90^{\circ }\right]$, there are 32,669 candidate directional information sets in a dictionary A.

Then, by checking the difference between the measured received signal and all candidate phases, the direction with the smallest difference should be selected. Here, the cost function, which is the criterion for determining the smallest phase difference, can exist in various forms [22]. In this paper, we utilize the cosine function as a cost function since it has the best performance among cost functions in [22], and it is designed as

(6) $$J_{\mathrm{CI}}\left(\hat{\gamma }\right)=\sum_{m=1}^{M}\cos\left[c_{m}\left(\hat{\gamma }\right)-r_{m}\right].$$

Interestingly, in (6), the direction with the smallest phase difference has the maximum value, and the direction with a difference of more than 90 degrees has a negative value due to the property of the cosine function. These properties help the direction-finding performance to improve even if some outliers caused by additive noise in the measured phase exist in arbitrary antennas.

Now, we explain the detailed description of the CI algorithm. The conventional CI algorithm simply consists of three-steps as follows. First, the phase of the received signal $r_{m}$ is measured, and candidate phases (4) are generated for (5). Then, the receiver calculates and stores the cost function as described in (6) for all candidate directional information. Finally, since $J(\hat{\gamma })$ fluctuates for all $\hat{\gamma }$, the receiver selects indices corresponding to *K* maximum peaks, which is the number of signal sources. After that, the direction finding of the received signal sources can be completed by replacing the selected indices with the corresponding candidate directional information in the dictionary A. This process can be summarized as in Algorithm 1.

**Figure 2:**
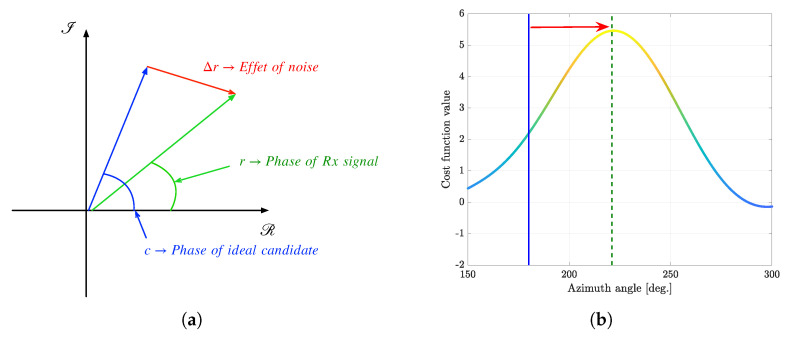
An example of the conventional CI-based DOA estimation due to additive noise (ideal DOA is 180${}^{\circ }$; estimated DOA is 196${}^{\circ }$). (**a**) Effects of additive noise on phase angle of complex signal. (**b**) Effects of additional noise on cost function value estimation.

Figure 2 shows a simple example explaining the direction-finding mechanism of the CI algorithm when a signal of a single source is received by a certain antenna. Specifically, Figure 2 visualizes the phase of the received signal on the complex plane, which is denoted as *r*. Here, assuming the received signal is incident at an azimuth angle of 180 degrees, the ideal phase for the corresponding direction is denoted as *c*. So, the difference between the phase of the received signal *r* and the ideal phase *c* can be viewed as the effect of additive noise, which is denoted as Δr. Since the effect of noise on the phase is related to the SNR, and the effect of Δr on each antenna occurs independently for all antennas, the CI algorithm can accurately estimate the direction if the SNR and the number of antennas are sufficient. In other words, in the case of low SNR and smaller number of antennas, the CI algorithm can significantly degrade direction-finding performance, as shown in Figure 2. Therefore, in these environments, another dimension to suppress the effects of noise might be needed as a solution for the performance improvement. With additional time-domain samples, in this paper, we address these problems in Section 4.
**Algorithm** **1** Correlative Interferometer for DOA estimation
 **Input:**
Directional information dictionary A, Received signal vector y, The number of signal sources *K*
 **Output:**
Estimated DOA Λ1:Initialization: Λ=⌀, Q=⌀2:**for **i=1,⋯,A** do**3:      **for** m=1,⋯,M **do**4:            $r_{m}=∠y_{m}$5:            $c_{m}\left({\hat{\gamma }}_{i}\right)=-\frac{2\pi }{\lambda }p_{m}\cdot s\left({\hat{\gamma }}_{i}\right)$6:      **end for**7:      $J_{\mathrm{CI}}\left({\hat{\gamma }}_{i}\right)=\sum_{m=1}^{M}\cos\left[c_{m}\left({\hat{\gamma }}_{i}\right)-r_{m}\right].$8:**end for**9:*Q*← Indices corresponding to *K* peaks in $J_{\mathrm{CI}}\left({\hat{\gamma }}_{i}\right),\;\forall i$.10:Λ←A(Q).11:**Return **Λ

## 4. Proposed Simultaneous Correlative Interferometer

In this section, we propose a novel simultaneous CI technique to jointly utilize spatial features in multiple time-domain samples. First of all, for the proposed simultaneous CI technique, the receiver collects *T* samples. Here, it is assumed that the direction of the incident signal sources does not change during the *T* sample period. Then, the received signal matrix $Y(\in C^{M\times T})$ for *T* time samples is expressed as

(7) Y=AX+W,

where Y consists of *T* columns in which the t(∈{1,⋯,T})-th column denotes the received signal vector $y_{t}\left(\in C^{M}\right)$ at *t*-th time, $X(\in R^{K\times T})$ denotes the concatenate transmitted signal matrix over *T* time samples, and $W(\in C^{M\times T})$ denotes the matrix of noise samples for *T* time samples where each column indicates the additive noise vector for each time sample. It is worth noting that the steering matrices A in both (1) and (7) are the same as each other since we assume that the DOA angles of multiple signal sources are not changed over multiple time samples in this paper.

Simultaneous CI technique is intended to efficiently utilize the difference between the phase of the received signal and ideal candidate phases over all time samples to average out the effects of noise. Hence, letting the *m*-th element of $y_{t}$ be $y_{m,t}$ and the phase of $y_{m,t}$ be $r_{m,t}$, i.e., $r_{m,t}:=∠y_{m,t}$, the temporal cost function $J_{t}\left(\hat{\gamma }\right)$ for an arbitrary directional information $\hat{\gamma }\in A$ at *t*-th sample can be defined as

(8) $$J_{t}\left(\hat{\gamma }\right)=\sum_{m=1}^{M}\cos\left[c_{m}\left(\hat{\gamma }\right)-r_{m,t}\right].$$

Finally, by adding the temporal cost functions for all samples, the cost function of simultaneous CI can be calculated as

(9) $$J_{\mathrm{SCI}}\left(\hat{\gamma }\right)=\sum_{t=1}^{T}J_{t}\left(\hat{\gamma }\right)=\sum_{t=1}^{T}\sum_{m=1}^{M}\cos\left[c_{m}\left(\hat{\gamma }\right)-r_{m,t}\right].$$

After that, DOA estimation is completed by finding *K* peaks in the cost function, same as the conventional CI method. The overall process of the simultaneous CI technique is summarized as Algorithm 2. The proposed simultaneous CI algorithm repeatedly performs phase comparison operation for *T* time samples. For example, when T=10, the proposed algorithm performs 10 phase comparisons. Then, the DOA angle of signal sources is estimated through the cost function by using all computed phases collectively, as shown in line (10) of Algorithm 2 in this paper. It is worth noting that the conventional CI algorithm just averages out multiple phases of the received signal during *T* time samples and exploits it only once for estimating DOA of signal sources.
**Algorithm** **2** Simultaneous CI for DOA estimation
 **Input:**
Directional information dictionary *A*, Received signal matrix Y, The number of signal sources *K*
 **Output:**
Estimated DOA Λ1:Initialization: Λ=⌀, Q=⌀2:**for **i=1,⋯,A** do**3:      **for** t=1,⋯,T **do**4:            **for** m=1,⋯,M **do**5:                  $r_{m,t}=∠y_{m,t}$6:                  $c_{m}\left({\hat{\gamma }}_{i}\right)=-\frac{2\pi }{\lambda }p_{m}\cdot s\left({\hat{\gamma }}_{i}\right)$7:            **end for**8:            $J_{t}\left({\hat{\gamma }}_{i}\right)=\sum_{m=1}^{M}\cos\left[c_{m}\left({\hat{\gamma }}_{i}\right)-r_{m,t}\right].$  9:       **end for**10:     $J_{\mathrm{SCI}}\left({\hat{\gamma }}_{i}\right)=\sum_{t=1}^{T}J_{t}\left({\hat{\gamma }}_{i}\right)$11:**end for**12:*Q*← Indices corresponding to *K* peaks in $J_{\mathrm{SCI}}\left({\hat{\gamma }}_{i}\right),\;\forall i$.13:Λ←A(Q).14:**Return **Λ

## 5. Comparison of MUSIC and CI-Based DOA Estimation Algorithms

**Figure 3:**
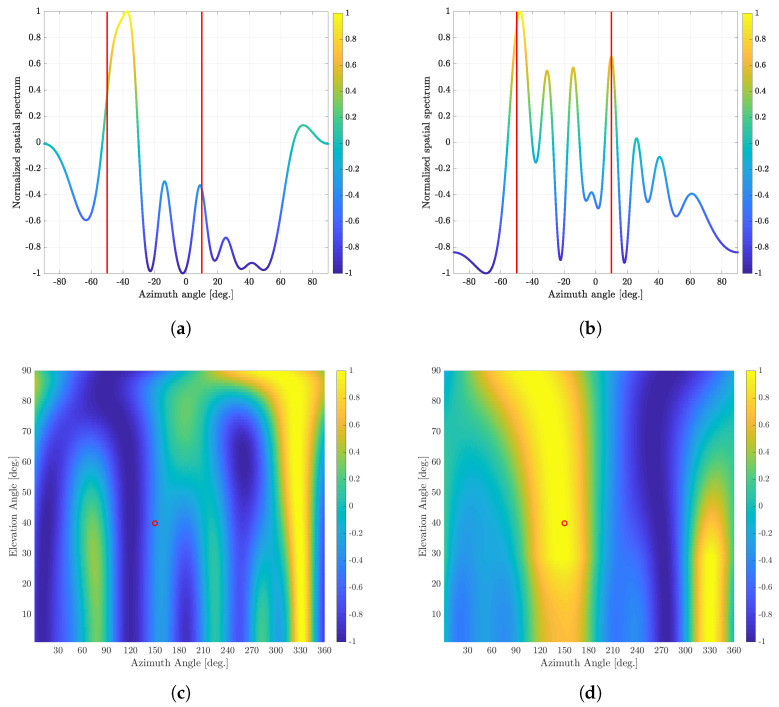
An example of normalized DOA spectra of the conventional MUSIC and CI algorithms. For a ULA receiver, there exist two signal sources with different azimuth DOA angles, which are equal to $-50^{\circ }$ and $10^{\circ }$. For a UCA receiver, there exists a single signal source whose azimuth and elevation DOA angles are equal to $150^{\circ }$ and $40^{\circ }$, respectively. (**a**) Normalized DOA spectrum of the conventional MUSIC algorithm with a ULA receiver. (**b**) Normalized DOA cost function value of CI algorithm with a ULA receiver. (**c**) Normalized 2D-DOA spectrum of conventional MUSIC algorithm with a UCA receiver. (**d**) Normalized 2D-DOA cost function value of CI algorithm with a UCA receiver.

In this section, the existing MUSIC and CI techniques are compared by using the DOA spectra, which indicate the normalized spatial spectrum for the MUSIC algorithm and the cost function value for the CI technique, respectively. Figure 3 shows the DOA spectra for both MUSIC and CI techniques. In this simulation, we also consider the ULA antenna structure at the receiver for providing clear comparison between MUSIC and CI algorithms. For the ULA-based receiver, we consider two signal sources with different azimuth (horizontal) DOA angles, which are $-50^{\circ }$ and 10${}^{\circ }$. On the other hand, for the UCA receiver, we consider a single signal source with azimuth DOA angle of $150^{\circ }$ and elevation DOA angle of 40${}^{\circ }$, i.e., ϕ = 150${}^{\circ }$, θ = 40${}^{\circ }$. The number of antennas in both ULA and UCA array structures are assumed to be 10, i.e., M=10. For both array structures, the SNR and the number of time samples are assumed to be −10 dB and 10, respectively.

When we use the ULA antenna structure at the receiver, both MUSIC and CI algorithms find two azimuth angles that yield the two largest DOA spectra or DOA cost function values in the range of azimuth angles, respectively, as DOA angles of two signal sources. On the other hand, when we use the UCA antenna structure at the receiver, both MUSIC and CI algorithms find a single tuple, consisting of azimuth and elevation angles, which yields the largest DOA spectrum or DOA cost function value in the two-dimensional DOA (azimuth and elevation angle) range, respectively, as a DOA angle of a single signal source. It is worth noting that the number of time samples may not be sufficiently large and the spatial correlation is not well-captured in the computed covariance matrix for the MUSIC algorithm in Figure 3. Thus, the resultant direction-finding performance of MUSIC algorithm is not very satisfactory. On the other hand, the conventional CI-based direction-finding technique finds the direction of received signals by directly comparing the phases of the received signal with reference dictionaries generated by assuming ideal phases of a certain signal direction. Thus, the CI-based direction-finding technique provides more accurate direction-finding performance compared with MUSIC algorithm for both ULA and UCA antenna structures, as shown in Figure 3. The conventional CI outperforms the conventional MUSIC when the number of time samples is small or the received SNR is low. In fact, obtaining enough time samples to rigorously analyze the spatial correlation characteristics of the received signals may not be *feasible* in practical dynamic environments, including military communication systems with anti-jamming/anti-spoofing functions, low earth orbit (LEO) satellite communication systems, and 5G/6G mobile communication systems with multiple-input multiple-output (MIMO) DOA beamforming. Hence, the CI technique may be appropriate for such practical dynamic environments due to its simple operation with direct phase comparison.

## 6. Simulation Results

In this section, we validate the effectiveness of the proposed simultaneous CI technique and compare it with both the conventional CI method and the classical subspace-based MUSIC method. In addition, as described above, we show that the multi-sample diversity of the proposed simultaneous CI technique improves the direction-finding performance even in the low-SNR regime.

For computer simulations, we set the carrier frequency of signals to 1542 MHz, which is equal to the carrier frequency of the Global Positioning System (GPS) over L1 band. A UCA with *M* antenna elements is considered, and the radius of the UCA is assumed to be λ/2, where λ represents the wavelength of the carrier frequency. Both UCA and ULA with M antenna elements are considered. In the UCA structure, the radius is assumed to be spaced at one degree for both the azimuth $\hat{\phi }\in \left(0^{\circ },360^{\circ }\right]$ and elevation angle $\hat{\theta }\in \left[0^{\circ },90^{\circ }\right]$. The detailed simulation parameters are summarized in Table 1.

**Figure 4:**
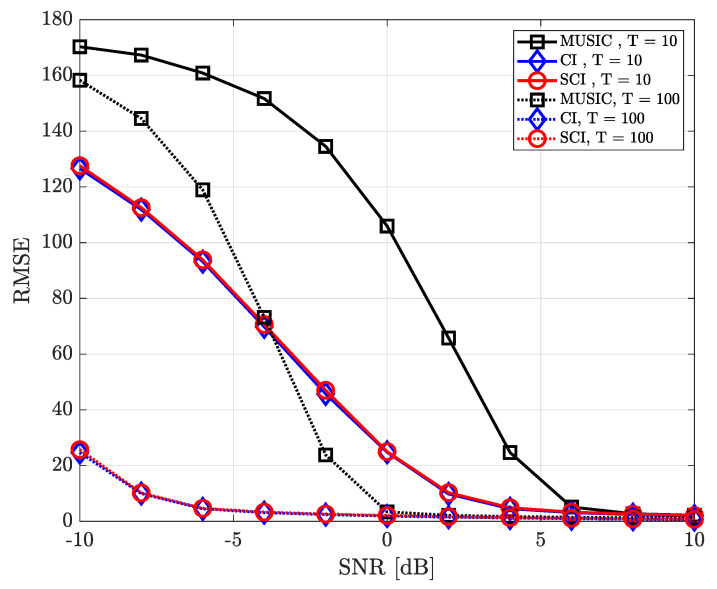
RMSE of DOA estimation performance for varying SNR when *K* = 1 and *M* = 10 at UCA receiver.

**Figure 5:**
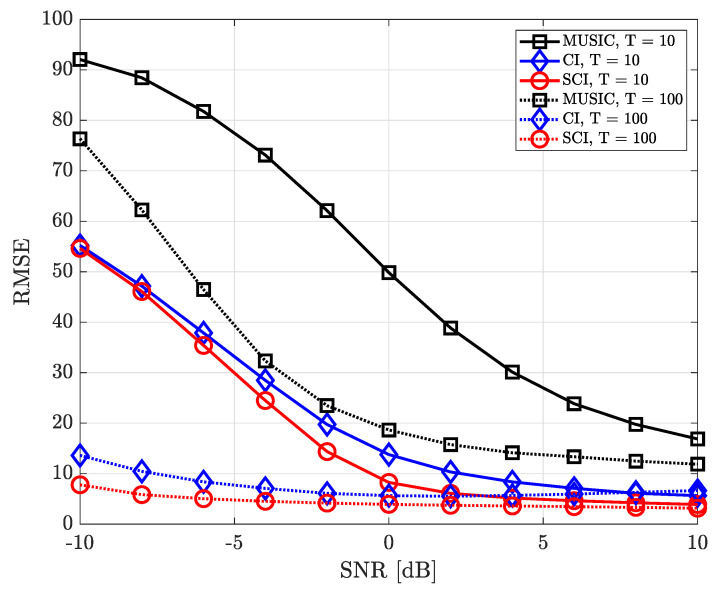
RMSE of DOA estimation performance for varying SNR when *K* = 2 and *M* = 10 at UCA receiver.

**Figure 6:**
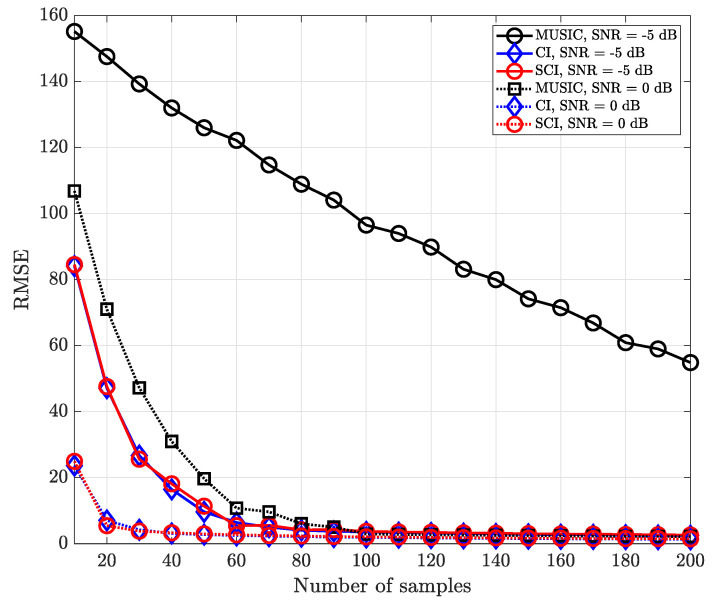
RMSE of DOA estimation performance for varying number of samples when K=1 and M=10 at UCA receiver.

**Figure 7:**
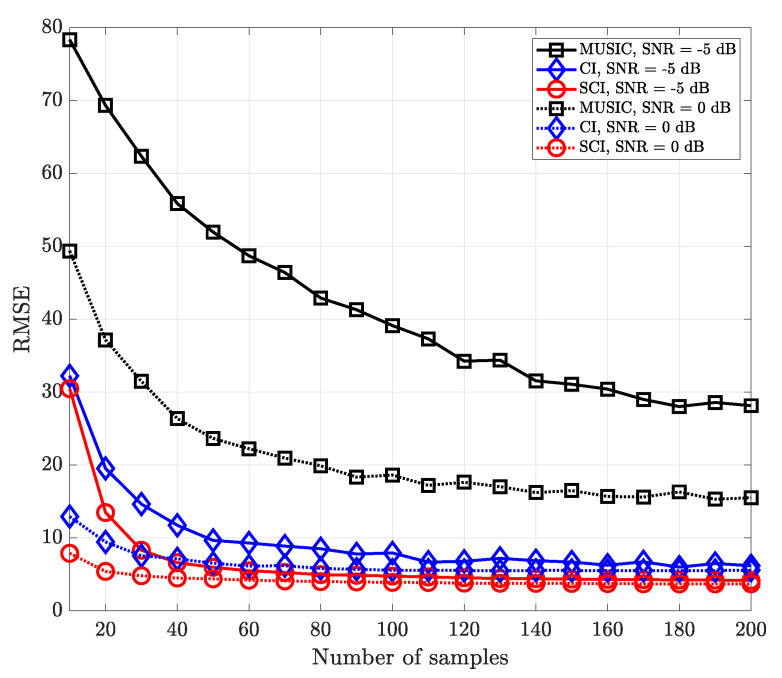
RMSE of DOA estimation performance for varying number of samples when K=2 and M=10 at UCA receiver.

**Figure 8:**
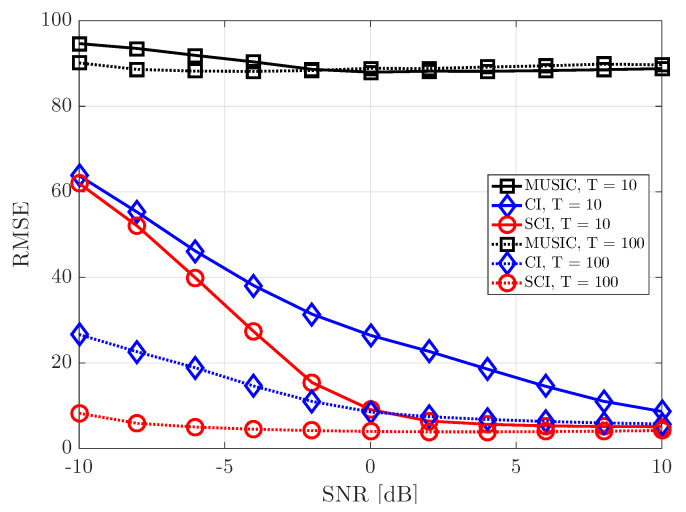
RMSE of DOA estimation performance for varying SNR when K=2 and M=5 at UCA receiver.

**Figure 9:**
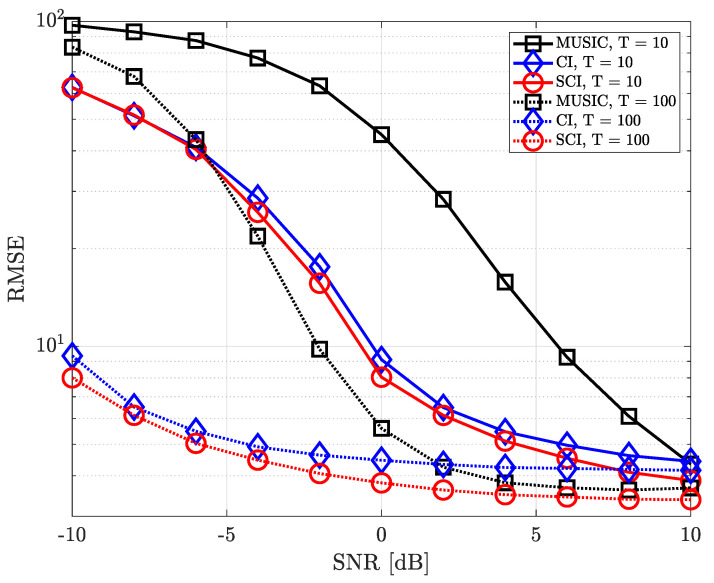
RMSE of DOA estimation performance for varying SNR when K=2 and M=20 at UCA receiver.

**Figure 10:**
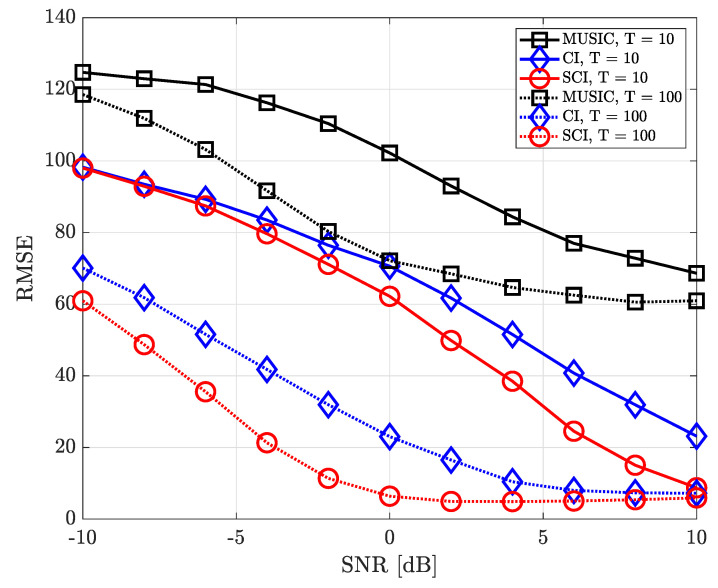
RMSE of DOA estimation performance for varying SNR when two signal sources are located in a similar direction at UCA receiver.

As a performance metric, the Root Mean Squared Error (RMSE) is considered to evaluate the direction-finding performance through Monte Carlo simulations. Specifically, when the azimuth and elevation angle estimates for the *k*-th source in the n∈{1,⋯,N} iteration are ${\hat{\phi }}_{k,n}^{*}$ and ${\hat{\theta }}_{k,n}^{*}$, respectively, the RMSE is defined as [30]

(10) $$\mathrm{RMSE}=\sqrt{\frac{1}{NK}\sum_{n=1}^{N}\sum_{k=1}^{K}\left[{\left({\hat{\phi }}_{k,n}^{*}-\phi_{k,n}\right)}^{2}+{\left({\hat{\theta }}_{k,n}^{*}-\theta_{k,n}\right)}^{2}\right]},$$

where $\phi_{k,n}$ and $\theta_{k,n}$ denote true azimuth and elevation angle of the *k*-th source in the *n*-th iteration, respectively. Figure 4, Figure 5, Figure 6, Figure 7, Figure 8, Figure 9 and Figure 10 show the RMSE of DOA estimation performance of the proposed simultaneous CI-based DOA estimation technique with UCA structure at the receiver. Figure 4 shows the RMSE performance of the simultaneous CI technique with UCA receiver according to SNR while considering a single source over multiple time-domain samples. In this simulation, Figure 4 considered DOA of signal sources $\phi =40^{\circ }$ and $\theta =10^{\circ }$, respectively. For the conventional CI method, the mean value of the received signal over all samples is used for DOA estimation. The RMSE of all DOA estimation techniques tends to decrease as the number of time-domain samples increases. In addition, it is observed that, for a single source, not only our proposed simultaneous CI but also the conventional CI outperforms the classical MUSIC at all SNR regions. This implies that for detecting a single source, the performance of the conventional CI method is sufficiently improved through simply averaging the received signals using multiple time-domain samples.

Figure 5 shows the RMSE performance considering two signal sourecs in which the azimuth and elevation angles of these two sources are set to $\phi =\{40^{\circ },180^{\circ }\}$ and $\theta =\{10^{\circ },5^{\circ }\}$, respectively. In such an environment, when estimating one DOA, the directional component of the other signal source may interfere and it makes the accurate direction finding hard. Nevertheless, it is confirmed that the proposed simultaneous CI technique yields better RMSE performance than the conventional CI technique and MUSIC method by jointly combining spatial features of signal components for each sample. In other words, it is verified that the multi-sample diversity effect efficiently works in all SNR regions.

Figure 6 shows the RMSE performance versus the number of time-domain samples in a single signal-source environment for various SNR values. Basically, it is observed that the CI-based DOA estimation methods outperform the subspace-based MUSIC algorithm at low-SNR regions. Likewise, in the case of a single signal source, the performances of the CI and SCI techniques are almost the same to each other since both methods exploit sufficient time-domain samples to reduce the effect of noise.

Figure 7 shows the RMSE performance for multiple signal sources in the same environment in Figure 6. As the number of samples increases, the multi-sample diversity improves the direction-finding performance, but the effect of the multi-sample diversity becomes saturated as the number of samples increases. It is worth noting that the proposed simultaneous CI technique significantly outperforms the MUSIC method regardless of the number of time-domain samples and operating SNR values.

Figure 8 shows the RMSE performance for varying SNR values when the number of UCA antenna elements is equal to 5 (M=5) and two signal sources exist (K=2). As the number of antenna elements in UCA decreases, the corresponding beam width becomes wider. Then, the DOA estimation performance becomes gradually deteriorated in both MUSIC- and CI-based DOA estimation techniques. Nonetheless, the conventional and proposed CI-based techniques perform better than the MUSIC-based DOA estimation technique and result in more accurate DOA information when SNR is sufficiently high, even in the case of a small number of time samples (T=10). Furthermore, the proposed simultaneous CI technique achieves the best DOA estimation performance among all the considered DOA estimation techniques. When a sufficient number of time samples are obtained (T=100), the proposed simultaneous CI technique achieves a very elaborate DOA estimation performance even in a low-SNR regime.

Figure 9 shows the RMSE performance of DOA estimation for varying SNR when the number of UCA antenna elements is equal to 20 (M=20) and two signal sources exist (K=2). In this figure, we assume a sufficient number of antenna elements for better DOA resolution with narrow beam width. When T=10, the MUSIC-based DOA estimation technique results in the good RMSE performance only in a high-SNR regime, while the proposed simultaneous CI-based DOA estimation technique results in a good RMSE performance even in a low-SNR regime. On the other hand, when T=100, the MUSIC-based DOA estimation algorithm performs better than the conventional CI-based DOA estimation technique in a high-SNR regime. It is worth noting that the proposed simultaneous CI-based DOA estimation technique results in the best performance regardless of the number of time samples and SNR values.

Figure 10 shows the RMSE of DOA estimation performance for varying SNR when two signal sources exist in a similar direction. In this simulation, two signal sources are located in directions of $\phi =\{40^{\circ },130^{\circ }\}$ and $\theta =\{10^{\circ },5^{\circ }\}$, respectively. As mentioned earlier, the DOA estimation performance of the MUSIC-based algorithm becomes significantly deteriorated in this situation since the spatial correlation between two received signals increases. However, the CI-based techniques result in robust DOA estimation performances even when signal sources are quite close to each other in terms of directions.

**Figure 11:**
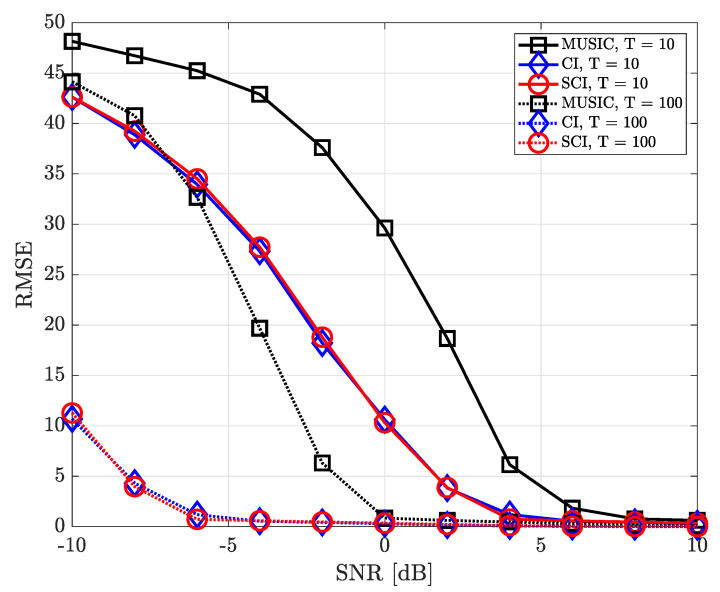
RMSE of DOA estimation performance with ULA structure according to SNR when a single signal source exists (K=1).

**Figure 12:**
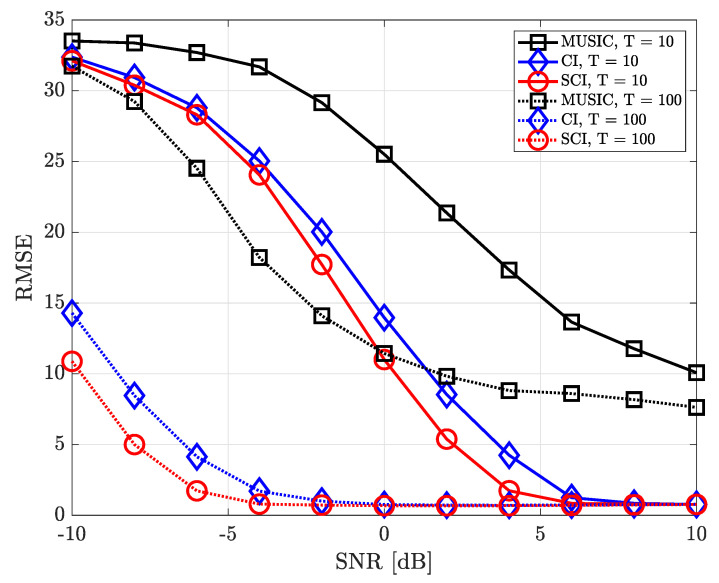
RMSE of DOA estimation performance with ULA structure according to SNR when two signal sources exist (K=2).

**Figure 13:**
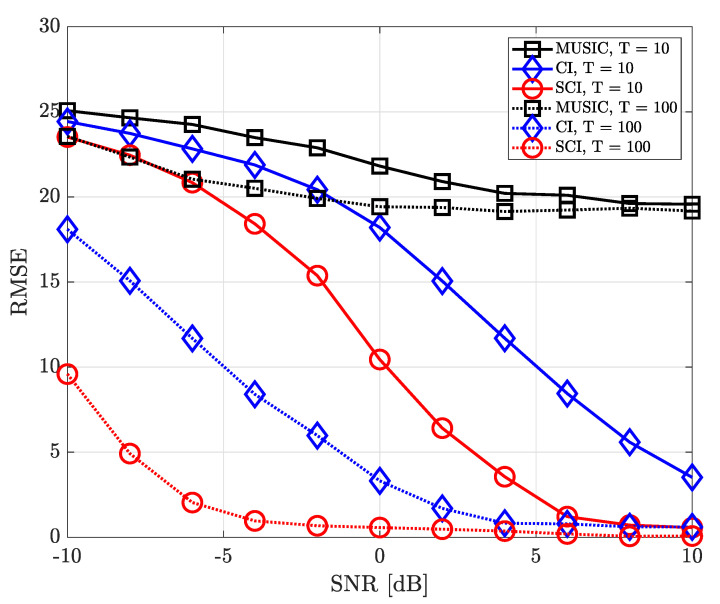
RMSE of DOA estimation performance with ULA structure according to SNR when three signal sources exist (K=3).

The proposed simultaneous CI-based DOA estimation technique can be applied to other antenna array structures, including ULA. Figure 11, Figure 12 and Figure 13 show the RMSE of DOA estimation performance of the proposed simultaneous CI-based DOA estimation technique with ULA structure at the receiver for varying SNR when the number of signal sources is equal to 1, 2, and 3, respectively. In the ULA structure at the receiver, the steering vector of the received signal is provided by

(11) $$a_{m}\left(\phi_{k}\right)=e^{-j\frac{2\pi }{\lambda }d\left(m-1\right)cos\left(\phi_{k}\right))},$$

where *d* denotes antenna spacing. Note that the ULA structure can estimate only one-dimensional DOA due to its fundamental physical constraint. Figure 11 shows the RMSE of DOA estimation performance of the proposed simultaneous CI-based DOA estimation technique with ULA structure at the receiver according to SNR when a single source exists. In this figure, the source is assumed to be located with the azimuth angle of $-40^{\circ }$, i.e., $\phi_{1}=-40^{\circ }$. In this figure, the CI-based techniques perform much better than the MUSIC algorithm, especially in a low-SNR regime, and the proposed simultaneous CI-based technique results in a very similar DOA estimation performance with the conventional CI-based technique. Figure 12 and Figure 13 show the RMSE values of DOA estimation performance of the proposed simultaneous CI-based DOA estimation technique when two and three signal sources exist, respectively. In Figure 12, we consider the following azimuth angles for two signal sources: ϕ = {−30${}^{\circ }$, 10${}^{\circ }$}. In Figure 13, we consider the following azimuth angles for three signal sources: ϕ = {−30${}^{\circ }$, 10${}^{\circ }$, 50${}^{\circ }$}. We show that the proposed simultaneous CI-based DOA estimation technique outperforms the conventional CI-based and MUSIC-based algorithms regardless of SNR values and the number of time samples. Interestingly, the performance gaps between the proposed simultaneous CI-based DOA estimation technique and the conventional techniques becomes large as the number of signal sources increases.

## 7. Conclusions

In this paper, we proposed a novel simultaneous Correlation Interferometer (CI) technique for improving Direction of Arrival (DOA) estimation performance, which effectively exploits multiple time samples and can be considered a generalization of the conventional CI technique. We first investigated the operation procedure of an existing CI algorithm in detail and discussed its technical challenges. As a novel solution, we proposed a simultaneous CI technique that allows the multi-antenna receiver to jointly combine the spatial characteristics of each time sample for improving direction-finding performance thanks to the multi-sample diversity effect. Through computer simulations, we showed that the proposed simultaneous CI technique significantly outperforms the conventional DOA estimation techniques with various array structures, Signal-to-Noise Ratio (SNR) values, and the number of time samples. It is worth noting that the proposed simultaneous CI technique has a better DOA estimation performance than classical MUSIC algorithms in terms of DOA estimation performance, especially with a small number of time samples and antenna elements. As a result, the proposed simultaneous CI-based DOA estimation algorithm can be effectively applied to various dynamic DOA estimation systems, such as 5G/6G wireless communication systems, radar systems, multiple antenna-based DOA beamforming systems, GNSS anti-spoofing/anti-jamming systems, military communication systems, etc. As further work, we plan to implement the proposed technique to consider its hardware complexity and feasibility.

## Figures and Tables

**Table 1:** Simulation parmeters.

Parameter	Value
Center frequency	1542 MHz
Wavelength	0.1944 m
Array structure	UCA, ULA
The number of antennas	5, 10, 20
Radius of UCA antenna structure	0.0972 m
Antenna spacing between of ULA structure	0.0972 m
Resolution for DOA estimation	$1^{\circ }$
Signal-to-Noise ratio	[−10:10] dB
The number of samples	[10:200]

## Data Availability

Not applicable.

## Abbreviations

The following abbreviations are used in this paper:

IIoT	Industrial Internet of Things
3GPP	3rd Generation Partnership Project
IEEE	Institute of Electrical and Electronics Engineers
DOA	Direction of Arrival
UAV	Unmanned Aerial Vehicle
V2X	Vehicle to Everything
GNSS	Global Navigation Satellite System
MLE	Maximum Likelihood Estimator
MUSIC	Multiple Signal Classification
IMLC	Iterative Maximum Likelihood Calibration
ESPRIT	Estimation of Signal Parameters via Rotational Invariance Techniques
CS	Compressed Sensing
CI	Correlative Interferometer
ULA	Uniform Linear Array
UCA	Uniform Circular Array
SNR	Signal-to-Noise Ratio
LOS	Line of Sight
AWGN	Additive White Gaussian Noise
LEO	Low Earth Orbit
MIMO	Multiple-Input Multiple-Output
GPS	Global Positioning System
RMSE	Root Mean Squared Error
